# Microstructure of CuCrZrV and ODS(Y_2_O_3_)-Cu Alloys After Neutron Irradiation at 150, 350, and 450 °C to 2.5 dpa

**DOI:** 10.3390/ma18071401

**Published:** 2025-03-21

**Authors:** Michael Klimenkov, Carsten Bonnekoh, Ute Jaentsch, Michael Rieth, Hans-Christian Schneider, Dmitry Terentyev, Koray Iroc, Wouter Van Renterghem

**Affiliations:** 1 Karlsruhe Institute of Technology (KIT), Institute for Applied Materials—Applied Materials Physics, 76021 Karlsruhe, Germany; 2 Karlsruhe Institute of Technology (KIT), Institute for Applied Materials—Mechanics of Materials and Interfaces, 76021 Karlsruhe, Germany; 3 Institute of Nuclear Materials Science, SCK CEN, 2400 Mol, Belgium; 4 Electron Microscopy for Materials Science, Department of Physics, University of Antwerp, 2000 Antwerp, Belgium

**Keywords:** CuCrZr, neutron irradiation, microstructure

## Abstract

In this study, the results of transmission electron microscopy (TEM) examinations of neutron-irradiated (2.5 dpa at 150 °C, 350 °C, and 450 °C) CuCrZrV and ODS(Y_2_O_3_)-Cu alloys are presented. These materials were developed for application as heat sink materials in fusion technology. This study includes TEM imaging and quantitative analysis of neutron radiation-induced defects such as dislocation loops and voids as well as the determination of the conditions for their formation. It was found that dislocation loops of a_0_½⟨110⟩ type form in both alloys at all irradiation temperatures. The formation of voids in CuCrZrV alloy is effectively suppressed. The neutron irradiation causes a redistribution of Cr, Zr, and V in the CuCrZrV alloy. A particular focus was on the investigation of the distribution of the transmutation products Ni and Zn. Ni tends to segregate at the Cr-rich clusters and forms a shell around them, while Zn is evenly distributed.

## 1. Introduction

Copper (Cu) and Cu-based alloys have been considered as heat sink materials for applications in fusion technology [1,2,3]. The progress in their development and application over more than five decades has been the subject of a number of review publications [2,4,5,6]. CuCrZrV and Cu-Y_2_O_3_ represent two different classes of reinforced Cu-based alloys, i.e., precipitation hardened (PH) Cu (CuCrZrV) and oxide-dispersion-strengthened (DS) Cu (ODS-Cu). These alloys combine good mechanical properties with high electrical and thermal conductivity.

The high strength of PH Cu-based alloys is achieved by a high density of nano-sized precipitates of second-phase particles. The strength of these alloys is influenced by factors such as particle size, number density, volume fraction, distribution, and the nature of the interphase boundaries. While these alloys can achieve significant strength, they are also susceptible to softening due to precipitates coarsening at intermediate to high service temperatures, which may induce recrystallization. Consequently, thermal processing can significantly affect the strength and conductivity of this class of Cu alloys.

DS Cu alloys contain a finely dispersion of nano-sized oxide particles, such as alumina, zirconia, hafnia, chromia, or yttria. This class of Cu alloys can be manufactured by either conventional powder metallurgy or by internal oxidation. The properties of these alloys are highly dependent on the type, size, volume fraction, and distribution of the dispersed phase, as well as the processing techniques. In contrast to PH Cu alloys, the finely dispersed oxide particles effectively prevent recrystallisation, so that the strength is maintained even at temperatures close to the Cu melting point. In addition, these oxide particles are insoluble in the solid matrix, which increases the stability of the alloy. Yttria (Y_2_O_3_) oxides are attractive dispersions due to their excellent thermal stability. The larger lattice mismatch at Y_2_O_3_/Cu interfaces than at Al_2_O_3_/Cu interfaces may explain its better high-temperature mechanical strength. Since these alloys are designed to operate under fusion conditions, knowledge of their response to neutron irradiation is of great importance to assess their operating limits. For this objective, radiation damage in Cu and Cu alloys has been intensively investigated in the past [7,8,9,10,11]. The mechanical properties of both examined alloys, before and after neutron irradiation, were reported in [12,13]. It was demonstrated that the CuCrZrV alloy, irradiated at 450 °C, exhibited significant softening. In contrast, the ODS-Cu alloy irradiated under the same conditions showed a reduction in strength, but no softening was observed. It has been shown that neutron irradiation causes the formation of stacking fault tetrahedra (SFT), dislocation loops, and voids, depending on the irradiation conditions. The formation of SFTs and dislocation loops typically occurs at low irradiation temperatures, while the formation of voids in pure Cu has been observed between 220 °C and 480 °C [8]. These defects are generally considered to be the reason for the degradation of the mechanical properties, in particular for the radiation hardening of the Cu-based alloys. It is also expected that Cu–Y_2_O_3_ alloys possess a better irradiation resistance due to the high defect sink strength of Y_2_O_3_ oxide interfaces, as already demonstrated for ODS steels [14,15].

Most of the neutron irradiation experiments and microstructural analyses have been carried out in the period between 1975 and 2005 [2,16,17]. Despite these studies, there are still some unanswered questions regarding the effects of neutron irradiation on the microstructure, which could be helpful in assessing the application limits of these materials. Recent developments in transmission electron microscopy (TEM) and sample preparation techniques open new technical possibilities for studying the effects of neutron irradiation on microstructure. The extremely sensitive EDX analysis can provide new insights into the redistribution of alloying and transmutation elements, even if these are present in very low concentrations. Previous studies have reported the effect of transmutation-induced Zn and Ni on macroscopic properties such as electrical and consequently thermal conductivity that is important for its application [18,19,20]. However, the microscopic distribution of the transmutation elements was not analyzed.

New Cu-based alloys also require the determination of their microstructural response to neutron irradiation. These factors necessitate the new microstructural investigations of neutron-irradiated Cu-based alloys. The present work also aims to gain knowledge on the radiation-induced defects formation and the diffusion processes in Cu-based alloys and how the irradiation temperature affects these processes. The mechanical properties before and after neutron irradiation have been reported in [12,21], while here the changes induced by irradiation in the microstructure are investigated. The present work aims to enhance the understanding of the formation of radiation-induced defects in CuCrZrV and Cu-Y_2_O_3_ alloys across a range of temperatures.

## 2. Materials and Methods

The investigated CuCrZrV alloy contains 0.922 wt.% Cr, 0.041 wt.% Zr, and 0.221 wt.% V. The as-delivered material was cold-rolled and then thermally treated at 500 °C for 2 h, followed by air cooling. The Cu-Y_2_O_3_ alloy was produced by Compound Extrusion Products (CEP) GmbH (Freiberg, Germany) following a proprietary technological process used similarly for commercial CEP DISCUP ODS(Al_2_O_3_)-Cu products. This experimental ODS-Cu alloy was mechanically alloyed with 0.70 wt.% Y_2_O_3_ and provided as a rod that was shaped by extrusion.

Neutron irradiation was performed in the BR2 (Belgian Material Test Reactor, Mol, Belgium) inside a fuel element in the radial position close to the reactor center and in the mid-plane horizontal position where the fast neutron (E > 0.1 MeV) flux is 4 × 10^14^ n/cm^2^/s at a power of 60 MW. The samples were encapsulated in a 1.65 mm steel tube that was filled with He. The gap between the samples and the tube was adjusted to achieve the target temperature following the thermal and neutronic calculations. The irradiation dose was calculated by MCNPX 2.7.0 to be ~2.5 dpa [22]. Irradiation was performed at 150 °C, 350 °C, and 450 °C. The irradiation dose was calculated by MCNPX 2.7.0 [12] and found to be 2.15 dpa, 2.5 dpa, and 2.55 dpa for the capsules with the samples irradiated at 150 °C, 350 °C, and 450 °C, respectively, as summarized in Table 1.

The preparation of the samples for TEM analysis was carried out employing a Scios (Thermo Fisher Scientific Inc. Waltham, MA, USA) focused ion beam (FIB) device. The microstructural examination was carried out using a Talos F200X (Thermo Fisher Scientific Inc. Waltham, MA, USA) transmission electron microscope (TEM) equipped with four energy-dispersive X-ray (EDX) detectors. The EDX detector resolution is specified by the manufacturer as ≤136 eV at Mn-Kα. The TEM images were acquired using a 16M CCD camera. The STEM-EDX maps were acquired using the Velox 3.15 software using 512 × 512 pixels and a spectral dispersion of 5 eV. ImageJ 1.53k software was used for statistical analysis of TEM and STEM images [23].

## 3. Results

### 3.1. Microstructures of Unirradiated CuCrZrV and ODS-Cu Alloys

**Figure 1:**
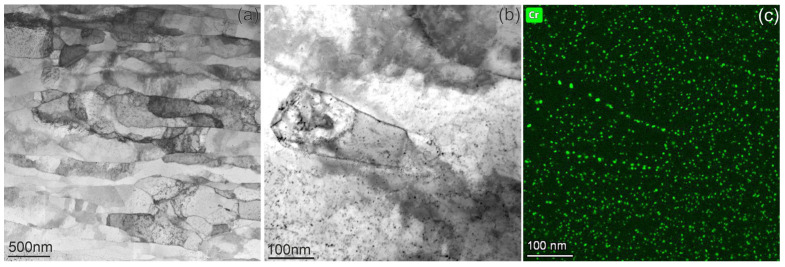
The microstructure of the unirradiated CuCrZrV alloy. Part (**a**) shows the grain structure, and parts (**b**,**c**) demonstrate the distribution of Cr particles.

**Figure 2:**
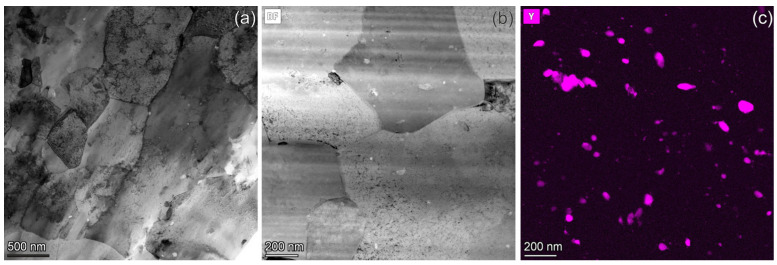
The microstructure of the unirradiated ODS-Cu alloy. Part (**a**) shows the grain structure, and parts (**b**,**c**) demonstrate the distribution of Y_2_O_3_ particles.

In order to determine the effects of neutron irradiation, the microstructures of both alloys were studied in the unirradiated state (Figure 1 and Figure 2). The CuCrZrV material consists of elongated grains with a thickness of 150–350 nm, which are orientated in the direction of the material deformation (Figure 1a). The analysis of the microstructure using scanning electron microscopy shows a homogeneous structure. Inside the grains, numerous defects a few nanometers in size are visible (Figure 1b). Figure 1c reveals that these defects are Cr-rich particles with an average size of ~2.5 nm. The particles are evenly distributed in the grains and often decorate the grain boundaries (Figure 1c). These particles are responsible for the hardening of CuCrZrV, and a modification of their morphology leads to an alteration of the mechanical properties [24]. The presence of Zr or V in these nanoscale precipitates was not detected. This might indicate a homogeneous distribution of these elements in the matrix. However, the very occasional occurrence of Cr/V intermetallic precipitates larger than 50 nm at low number density (~10^18^ m^−3^) was observed.

The grains in the Cu-Y_2_O_3_ alloy have a size in the range of 1 µm to 3 µm and usually contain a dislocation network with ~5 × 10^14^ m^−2^ density inside (Figure 2a). The Y-map that demonstrates the distribution of ODS particles in Figure 2c was obtained from the area shown in the Figure 2b. The particle size ranges from a few nanometers to 100 nm. Their number density was measured to be ~7 × 10^20^ m^−3^. However, the particles are inhomogeneously distributed.

**Figure 3:**
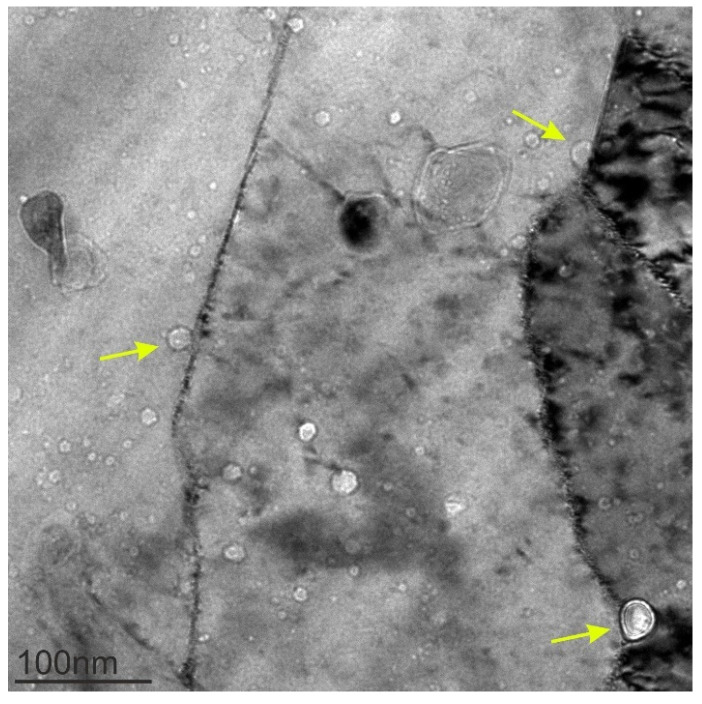
BF TEM image showing ODS particles and voids in the Cu-Y_2_O_3_ alloy. The ODS particles that are located on the grain boundaries are marked by yellow arrows.

It was found that the Cu-Y_2_O_3_ alloy already possessed spherical voids in the as-received state (Figure 3). The voids next to the ODS particles are visible with bright contrast in the bright-field TEM images, which were taken with a slight underfocus. Under these imaging conditions, the voids are difficult to differentiate from the ODS particles as they have a similar shape and contrast. Investigations using EDX mapping enable a clear differentiation and confirm the existence of voids with a size up to 20 nm. Some of them are located at the grain boundaries, where they presumably contribute to the pinning of grain boundaries.

### 3.2. Microstructure of the Irradiated CuCrZrV Alloy

**Figure 4:**
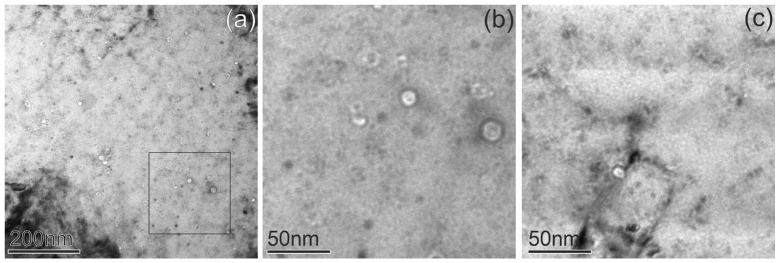
BF TEM images of voids in CuCrZrV alloys irradiated at 350 °C (**a**,**b**) and 450 °C (**c**).

The formation of voids in the CuCrZrV alloy was detected only at the 350 °C and 450 °C irradiation temperatures (Figure 4). The voids appear irregularly distributed with a very low density. At 350 °C, the voids are often located on the Cr-rich particles, and only a few of them have a spherical shape (Figure 4b).

**Figure 5:**
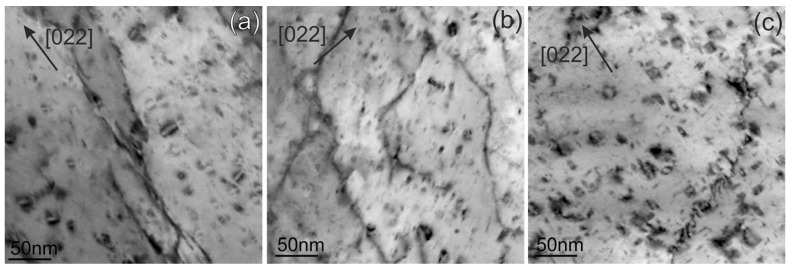
STEM-DF images of dislocation loops in CuCrZrV alloys irradiated at 150 °C (**a**), 350 °C (**b**), and 450 °C (**c**). The directions of the ***g***-vectors are indicated in the images with arrows.

Figure 5 shows examples of dislocation loops in CuCrZrV obtained in the dark field scanning TEM (DF-STEM) mode at all irradiation temperatures. The loops mostly have a size in the range of 7 nm to 20 nm for all temperatures; however, individual loops can reach a size of up to 50 nm. All dislocation loops have an a_0_½⟨110⟩ Burgers vector, as demonstrated in the next section. The formation of faulted loops with an a_0_1/3⟨111⟩ Burgers vector was not observed. Statistical data on dislocation loops and voids, including the average size and number density, as well as the void swelling derived from the TEM results, are listed in Table 2.

**Figure 6:**
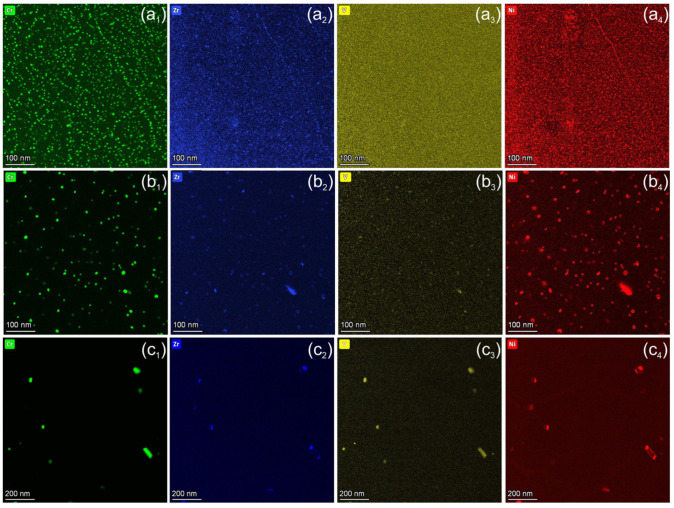
Elemental maps showing distribution (from left to right) of Cr, Zr, V, and Ni obtained from CuCrZrV alloys irradiated at 150 °C (**a_1_**–**a_4_**), 350 °C (**b_1_**–**b_4_**), and 450 °C (**c_1_**–**c_4_**).

**Figure 7:**
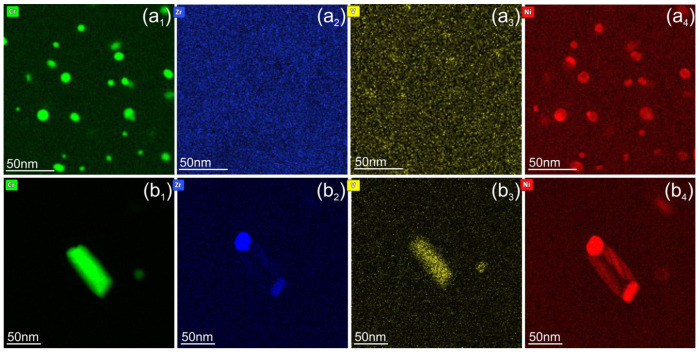
Elemental maps showing the distribution of Cr, Zr, V, and Ni obtained from CuCrZrV alloys irradiated at 350 °C (**a_1_**–**a_4_**) and 450 °C (**b_1_**–**b_4_**).

Figure 6 and Figure 7 show the effects of neutron irradiation and temperature on the distribution of alloying elements such as Cr, Zr, V, and transmutation-induced Ni. At 150 °C, the average size of Cr-rich precipitates increases from 2.7 nm to 3.3 nm compared to the unirradiated state (Figure 6a_1_). A weak Zr and Ni segregation at the particles could be detected (Figure 6a_1_,a_4_). A remarkable coarsening of Cr particles was detected after irradiation at 350 °C, where the average size increased to 8.3 nm, while their density is reduced by a factor of 5. For the sample irradiated at 450 °C, large complex Cr-rich particles were observed. Cr and V form particles with sizes of 20–150 nm, while Zr and Ni form a shell around these precipitates Figure 6c_1_–c_4_.

Ni and Zn were not present in the as-delivered Cu alloys and originate from ^63^Cu isotopes (about 70% existence in natural Cu) via the nuclear reaction ^63^Cu + n → ^64^Cu and subsequent β^+^ or β^−^ decay [25]. In the investigated materials, the Ni content is 0.6 ± 0.2%, as measured by quantification of the EDX spectra. Radiation-induced diffusion of Ni and Cr was observed at 150 °C. The size of the Cr-rich particles increases slightly from 2.7 nm to 3.5 nm, and a weak Ni segregation takes place on the particles (Figure 6a_4_). At 350 °C, Ni segregates and forms a shell around the Cr precipitates (Figure 7a_4_). Irradiation at 450 °C leads to the dissolution of finely dispersed Cr particles and the formation of complex precipitates up to 150 nm in size. These complex particles typically consist of two phases: Cr-V and Zr-Ni intermetallic phases. The Zr-Ni phase forms a shell or small particles on the surface of a core consisting of the Cr-V phase (Figure 7(b_2_,b_4_)). In addition to segregation on Cr-rich particles, Zr and Ni form particles of 20–30 nm in size, which are preferentially located at grain boundaries (Figure 6c_2_,c_4_). The formation of pure Ni particles was not observed. A quantitative analysis shows that approximately 70–80% of the Ni remains in the solid solution and only a small fraction segregates on the precipitates. For this objective, the Ni concentration was measured over a large area of approx. 2 µm^2^ and compared with the spectra taken from the area between the particles. At 450 °C, the Ni fraction in the solid solution is about 50%.

**Figure 8:**
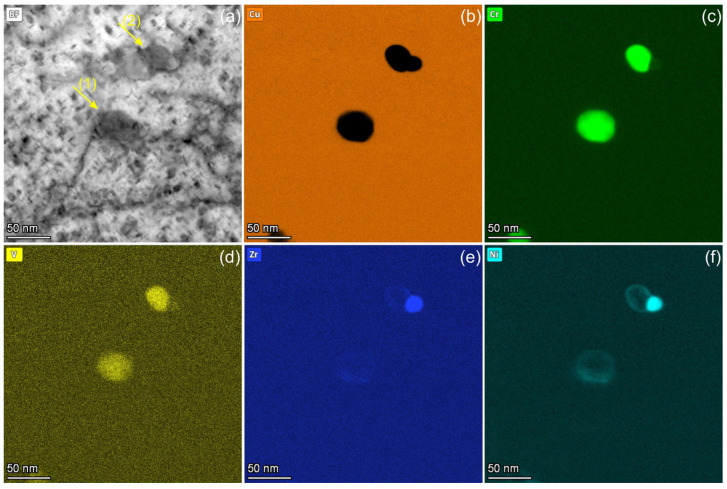
Elemental maps showing the composition of two precipitates marked in a BF image (**a**) and corresponding elemental maps of Cu (**b**), Cr (**c**), V (**d**), Zr (**e**), and Ni (**f**) obtained from the CuCrZrV alloy irradiated at 450 °C.

The analytical study presented in Figure 8 shows two particles with a size of approximately 50 nm, which were formed during irradiation at 450 °C. These particles are marked by arrows in Figure 8a. Particle (1) consists of 96% Cr, 1.5% V, and 3% Ni (at.%) (Figure 8c,d,f). Particle (2) comprises two distinct regions: one with a composition similar to particle (1), while the other contains 47% Zr and 53% Ni (at.%) (Figure 8e,f). Since the particles are embedded in a Cu matrix, the presence of Cu inside them, if any, cannot be definitively determined.

**Figure 9:**
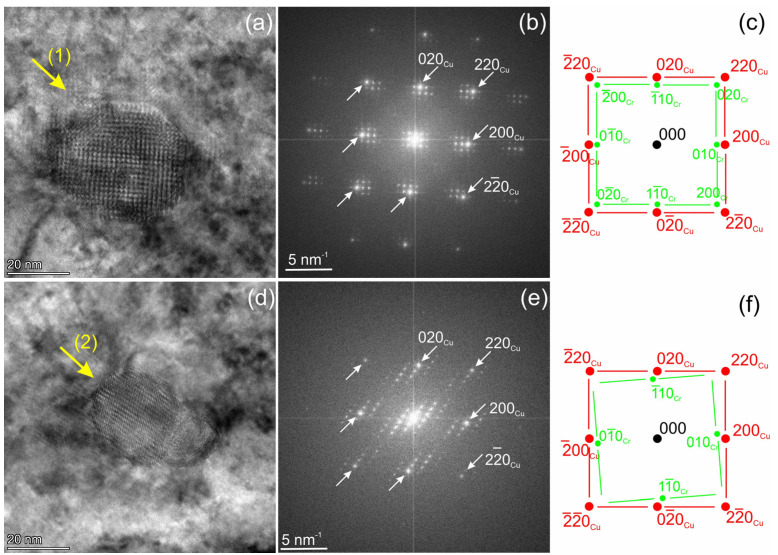
HRTEM images of the particles numbered (1) and (2), investigated in Figure 8, are shown in parts (**a**,**d**). The corresponding FFT images and structural analysis are presented in parts (**b**,**c**) and (**e**,**f**). Reflections from the particles (Cr) and the matrix (Cu) are provided in the images.

These two particles were analysed using high-resolution transmission electron microscopy (HRTEM) (Figure 9a,d). The corresponding fast Fourier transform (FFT) images and diffraction simulations are shown in Figure 9b,e and c,f, respectively. The Cu matrix is oriented with the [001] zone axis. The reflections of the Cu matrix are marked with arrows in the FFT images. Both Cr precipitates are also oriented with the [001] zone axis ([001]_Cu-fcc_ ∥ [001]_Cr-bcc_), showing a rectangular diffraction pattern. In the image plane, particle (1) shows an exact orientation to the Cu matrix [100]_Cu-fcc_ ∥ [110]_Cr-bc_ (Figure 9b,c). Due to the close interatomic distances, d_002Cu_ = 0.180 nm and d_011Cr_ = 0.204 nm, a Moiré patterns of 1.5 nm periodicity should appear at this orientation. Particle 1 exhibits two-dimensional rectangular Moiré fringes with a periodicity of 1.6 nm. Moiré fringes appear in the FFT image (Figure 9b) as a rectangular, low-intensity pattern surrounding the Cu reflections. Numerous additional low-intensity reflections next to Cu reflections are artefacts caused by double diffraction (Figure 9b).

As demonstrated in Figure 9e,f particle 2 has an deviation of 4.5° to the exact [100]_Cu-fcc_ ∥ [110]_Cr-bcc_ orientation. This results in a Moiré fringe periodicity of 1.25 nm, and the fringes do not form a rectangular pattern. The parts of particle 2 with a ZrNi composition (Figure 8e,f) appears to be in an amorphous state. No reflections additional to those of the Cu matrix were detected in this phase.

**Figure 10:**
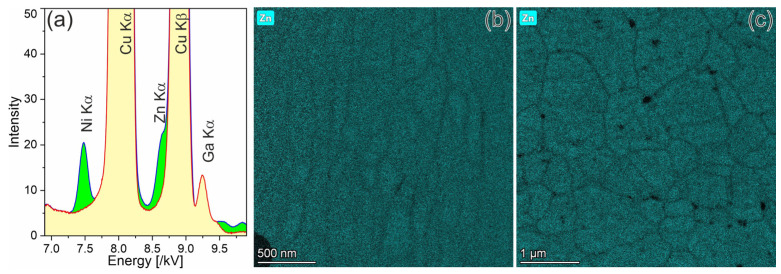
Analytical investigations of the distribution of Zn. Part (**a**) demonstrates the EDX spectra from unirradiated and irradiated CuCrZrV alloys, whereas parts (**b**,**c**) show the Zn distribution in the CuCrZrV and Cu-Y_2_O_3_ alloys, respectively.

As the inventory calculations for the neutron irradiation of Cu show, Zn is also formed in a 2/3 ratio to Ni [25]. Its presence in both alloys was also confirmed by EDX analysis (Figure 10a). The spectrum from the CuCrZrV alloy irradiated at 350 °C (green) was normalized by the intensity of Cu-Kα line and superimposed with that of the non-irradiated CuCrZrV alloy (yellow). In both cases, the spectra were obtained from the uniformly illuminated areas of ~2 µm^2^ that includes several grains. This procedure should ensure that the measured concentrations represent an average value for the samples and are not affected by the presence of larger inclusions. The spectrum of the irradiated material clearly shows the presence of 0.6 ± 0.2% Ni and 0.4 ± 0.15% Zn. The Zn-Kα EDX line is visible as a pre-shoulder of the Cu-Kα line. In contrast to Ni, which is attached to Cr particles (Figure 7), Zn does not segregate on any kinds of defects, particles, or grain boundaries. EDX mappings show that Zn is slightly depleted at the grain boundaries. This is valid for both the CuCrZrV (Figure 10b) and Cu-Y_2_O_3_ (Figure 10c) alloys and for all irradiation temperatures.

### 3.3. Radiation Damage in the ODS-Cu Alloy

**Figure 11:**
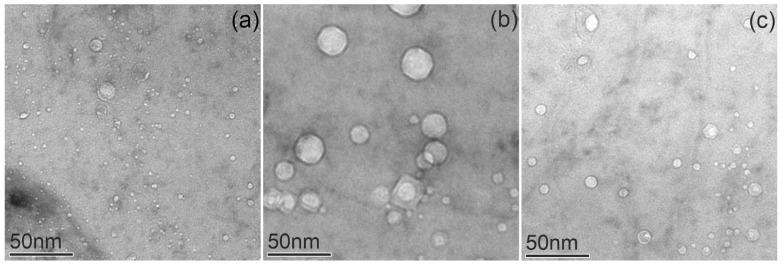
Bright-field TEM images showing voids in Cu-Y_2_O_3_ irradiated at 150 °C (**a**), 350 °C (**b**), and 450 °C (**c**).

In contrast to CuCrZrV, the formation of voids in the Cu-Y_2_O_3_ alloy took place at all studied irradiation temperatures. BF-TEM images taken from the 150 °C, 350 °C, and 450 °C samples are shown in Figure 11. It is evident that during neutron irradiation at 350 °C, the voids formed are significantly larger and have a lower number density compared to those formed at 150 °C and 450 °C. The void distributions show pronounced local differences in the size and number density. After 150 °C irradiation, voids of a few nanometers in size often decorate the grain boundaries (Figure 11a).

**Figure 12:**
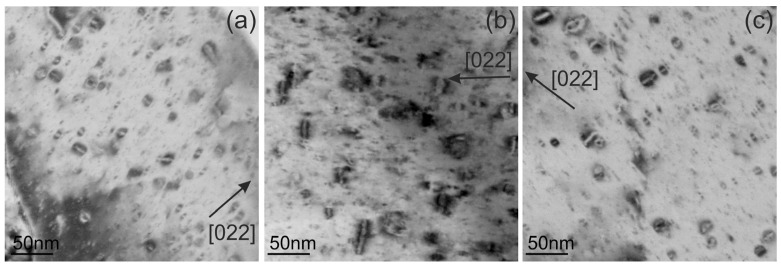
DF-STEM images with inversion contrast of dislocation loops in the ODS-Cu alloys irradiated at 150 °C (**a**), 350 °C (**b**), and 450 °C (**c**). The directions of the *g*-vectors are indicated in the images with arrows.

Dislocation loops observed in ODS-Cu irradiated at all three temperatures are shown in the DF-STEM images in Figure 12. The loops mostly have a size in the range from 8 nm to 25 nm for all temperatures; however, individual loops can reach a size of up to 50 nm. Statistical data on the dislocation loops and voids, including the average size, number density, and void swelling are listed in Table 3.

**Figure 13:**
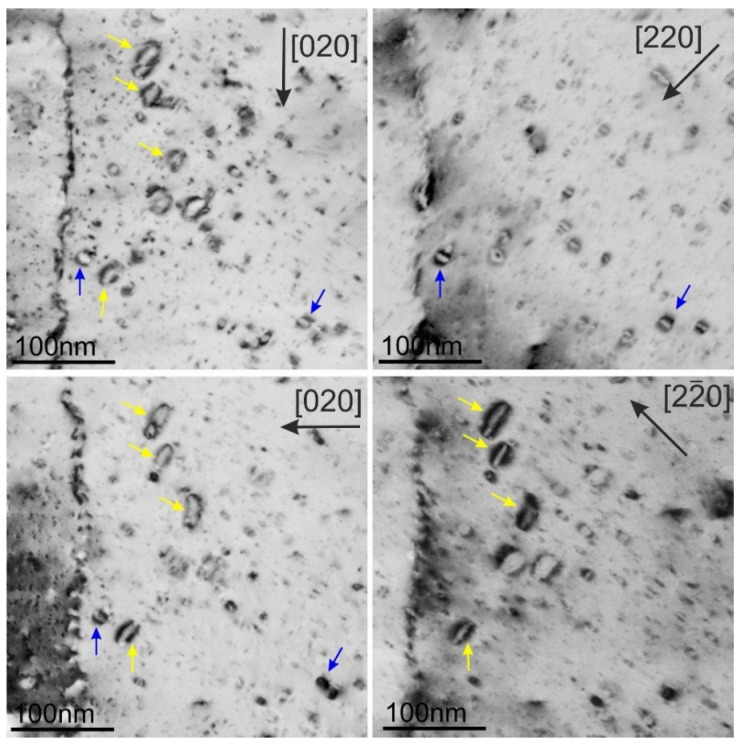
DF-STEM images of the same region obtained with four different *g*-vectors near the [100] zone axis. The direction of the *g*-vectors is indicated in the images. As an example, blue and yellow arrows indicate the loops with Burgers vectors of a_0_½[1$\bar{1}$0] and a_0_½[110] respectively.

The determination of the Burgers vector of the dislocation loops in the ODS-Cu alloy irradiated at 450 °C was performed using weak-beam DF imaging (WBDF) near the [100] zone axis (Figure 13). The applied method for materials with fcc structure, based on the visibility criterion of dislocations, is described by Xiu et al. [26]. The images of dislocations in a grain that was oriented near the [001] zone axis were obtained using [220], [2$\bar{2}$0], [002], and [200] g-vectors. All visible loops are of an a_0_½⟨110⟩ type. The loops with a_0_½[110] and a_0_½[$\bar{1}$10] Burgers vectors are marked correspondingly with yellow and blue arrows in the images. The formation of loops with an a_0_1/3⟨111⟩ Burgers vector was not detected. The analysis of dislocation loops at other irradiation temperatures and in CuCrZrV alloys shows identical results: all loops have a Burgers vector of an a_0_½⟨110⟩ type, and the formation of defective dislocation loops with an a_0_1/3⟨111⟩ Burger vector was not detected.

**Figure 14:**
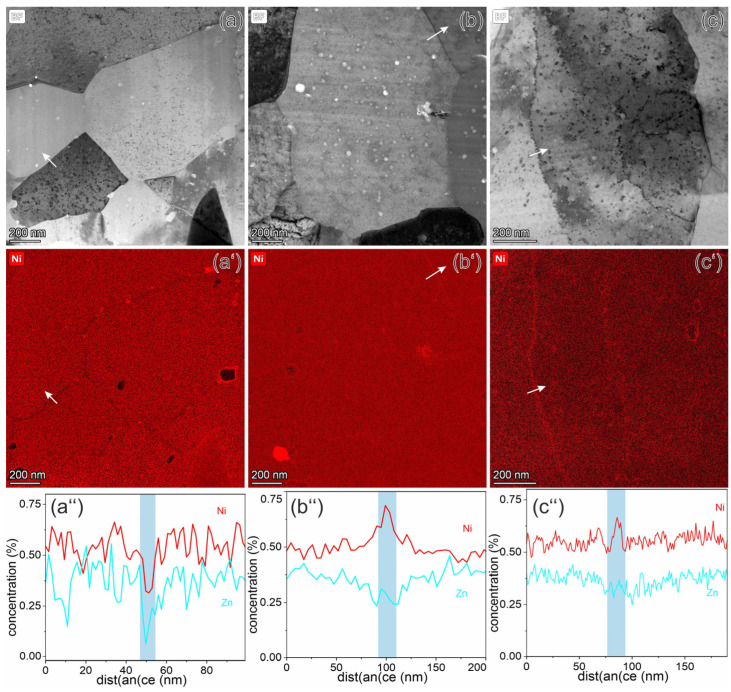
Analytical investigation of ODS-Cu alloys irradiated at 150 °C (**a**), 350 °C (**b**), and 450 °C (**c**). Parts (**a**–**c**) demonstrate the grain structure of the investigated area, and Ni maps of these areas are presented in parts (**a’**–**c’**), whereas the intensity profiles across marked lines of Ni and Zn are shown in parts (**a**″–**c**″). The positions of grain boundaries are highlighted in blue.

Analytical investigations show that the Y_2_O_3_ particles in Cu remain stable under neutron irradiation, as was already demonstrated for ODS steels [15]. There were no significant differences in the distribution of Y_2_O_3_ particles in comparison with the distribution in the as-supplied material (Figure 2). Zn is also homogeneously distributed in the matrix, with a very low depletion (~0.15 at.%) at the grain boundaries at all irradiation temperatures (Figure 10). The thickness of the Zn depleted layer on the grain boundaries increases with irradiation temperature from 10 nm at 150 °C to 40 nm at 450 °C, as shown in the intensity profiles across the grain boundaries (Figure 14).

In the irradiated ODS-Cu samples, Ni is homogeneously distributed in the matrix, although minor depletion and segregation effects at the grain boundaries were observed. At 150 °C, Ni depletion and formation of nanoscale Ni-containing precipitates along grain boundaries were observed (Figure 14a–a″). However, at 350 °C and 450 °C the Ni mapping shows a weak (0.5–0.75 at.%) enrichment (Figure 14b–b″,c–c″). This is demonstrated by the Ni profiles across the grain boundaries in the marked positions (Figure 14a″,b″,c″). Weak Ni segregation was also observed on some ODS particles. Voids, however, do not serve as segregation sites—neither for Ni nor Zn.

## 4. Discussion

The impact of neutron irradiation on the microstructure of metallic materials can be classified by two effects: the formation of structural lattice defects such as voids and dislocation loops and the radiation-induced decomposition/segregation/depletion of alloying (or transmutation-induced) elements. This can lead to the modification of pre-existing precipitates and the formation of new ones, resulting in a deterioration of mechanical and physical properties. In the case of Cu-based alloys, Ni and Zn are created by β^+^ or β^−^ decay from ^64^Cu during the irradiation [25].

TEM analyses of the neutron-irradiated CuCrZrV and ODS-Cu alloys show that voids formed in the ODS-Cu alloy at all irradiation temperatures, whereas void formation in CuCrZrV was strongly suppressed (Figure 4 and Figure 9). Dislocation loops show a similar structure in both materials (Figure 5 and Figure 10, Table 2 and Table 3). The Burgers vector was determined according to the method described in [26], in the orientation close to the [001] zone axis. This orientation enables reliable determination of the Burges vector and differentiation between perfect loops of the ½⟨110⟩ type and faulted loops of the 1/3⟨111⟩ type. The analysis proves that all observed loops have a perfect structure with an a_0_½⟨110⟩ Burger’s vector (Figure 13).

Irradiation-induced void swelling in ODS-Cu exhibited its highest value at 350 °C, while the swelling was significantly lower at 150 °C and 450 °C. (Table 3). This is consistent with the measurements of void swelling for neutron-irradiated pure Cu reported by Zinkle & Farrell [8]. The formation of voids in their work was observed in the irradiation temperature range between 200 °C and 450 °C. The swelling, measured by TEM, reached a maximum of ~0.5% at temperatures between 280 °C and 350 °C. The differences found in this work are related to void formation at low irradiation temperatures. Contrary to our results, the authors of [26] reported that voids were missing in pure Cu irradiated at 180 °C, whereas in the present study, voids in Cu-Y_2_O_3_ were detected at 150 °C with 0.15% swelling.

The high swelling tendency of Cu-Y_2_O_3_ that was observed in this work is most likely due to the use of boron (B) for deoxidation purposes. In contrast to CuCrZrV, the studied Cu-Y_2_O_3_ alloy is practically free of elements with high Gibbs energies of formation for oxide formation. Additional care must therefore be taken to ensure resistance to hydrogen reaction embrittlement (hydrogen sickness). However, natural boron consists of about 20% of the isotope ^10^B that releases helium under the flux of free neutrons by a ^10^B (n, α) ^7^Li reaction. In their work on two ODS(Al_2_O_3_)-Cu alloys, which were manufactured using the internal oxidation method, Fabritsiev et al. [27] pointed out the high swelling rate of the variant containing boron, while for the boron-free alloy no swelling was detected. The same relation has been reported by Zinkle [7] for neutron-irradiated B-doped and B-free ODS(Al_2_O_3_)-Cu (Glidcop) alloys. Therefore, conclusions on the swelling resistance of ODS(Y_2_O_3_)-Cu alloys must be postponed until materials using a different deoxidizer have been investigated.

As was shown above, in the ODS-Cu alloy, voids were already present in the non-irradiated condition (Figure 3). As was demonstrated previously, mechanical alloying with a subsequent HIPing procedure could lead to the formation of nano-sized bubbles inside the material [28]. After neutron irradiation at 150 °C, their number density increased by ~40 times, leading to the void swelling of 0.17% (Table 3). As was demonstrated by Zinkle & Li, void formation in Cu can be affected by various gaseous impurities such as oxygen or helium [29,30]. It was shown that the presence of oxygen enhances void nucleation/growth during neutron irradiation [29].

In CuCrZrV, voids were only detected after irradiation at 350 °C and 450 °C (Figure 4). They were inhomogeneously distributed, and their total swelling was negligible compared to the ODS-Cu alloy.

An effect of neutron irradiation on the Y_2_O_3_ particles in the ODS-Cu alloy was not found. However, due to the broad size range and the local variations in the distribution of the particles, a small modification in size/density would be difficult to observe with the aid of TEM images. In the CuCrZrV, however, a clear coarsening of finely dispersed Cr precipitates was detected. At 150 °C, the precipitate size just slightly increased, whereas at 350 °C, the increase of the precipitate size was remarkable, and the number density decreased by one order of magnitude (see Table 2). At 450 °C, all alloying elements formed large complex precipitates with a low number density. Their contribution to the strength of the material should be remarkably reduced. The similar effect of neutron irradiation on the Cr in CuCrZr alloys was reported in ref. [31], where the number density of Cr precipitates was reduced after the irradiation at 100 °C.

As is known, the neutron irradiation of Cu leads to the formation of Ni and Zn by β^+^ and β^−^ decay in the relation 62.9%/37.1% [32]. Our EDX analysis has proven the presence of ~0.6% Ni and ~0.4% Zn caused by neutron irradiation (Figure 10a). These two elements are soluble in Cu up to a concentration of about 38 at.% and with full solubility, respectively [33]. Therefore, it can be assumed that they are homogenously distributed in the material. Up to now, no microstructural studies examining the segregation or accumulation of Ni or Zn on grain boundaries, defects or precipitates in Cu are available. However, the presence of Ni or Zn was proven by a measurable reduction in electrical conductivity [9]. This should, therefore, also have a negative effect on the thermal conductivity.

It the present work, we were able to screen the distribution of Ni and Zn at the nanoscale level. In the CuCrZrV alloy, Ni was found to segregate on the Cr precipitates, forming a shell around the Cr rich phase (Figure 6). At 450 °C, a shell of NiZr intermetallic phase forms around the CrV precipitates (Figure 7). It is also found that at 350 °C about 30% of Ni and at 450 °C about 80% of Ni remains in solid solution. Zn is homogeneously distributed within the grains and is depleted near the grain boundaries in both the CuCrZrV and Cu-Y_2_O_3_ alloys (Figure 10b).

The analytical and HRTEM analyses show that Cr precipitates formed at 450 °C exhibit a bcc structure (Figure 8 and Figure 9). The orientation relationship was found to be close to the Bain orientation ([001]_Cu-fcc_ ∥ [001]_Cr-bcc_, [100]_Cu-fcc_ ∥ [110]_Cr-bcc_) [34]. This orientation has not been previously reported for Cr precipitates in the Cu matrix. As demonstrated, the coarsening of Cr precipitates in Cu during thermal treatment leads to the formation of particles with Nishiyama–Wassermann (NW) and Kurdjumov–Sachs (KS) orientations [35].

In the ODS-Cu alloy, Ni is homogeneously distributed within the grains. Only a very small fraction segregates at larger precipitates and grain boundaries. Segregation at structural defects such as dislocation loops or voids was not observed (Figure 14). After irradiation at 150 °C, Ni forms small precipitates at the grain boundaries. Such spatial distribution indicates preferential diffusion along the grain boundaries. At 350 °C and 450 °C, Ni atoms formed a week enrichment at the grain boundaries. It can be concluded that the spatial distribution of Ni is influenced by the presence of other alloying elements present in the studied alloys.

## 5. Conclusions

This publication is focused on TEM analysis of defects in CuCrZrV and ODS-Cu alloys irradiated at 150 °C, 350 °C, and 450 °C to a damage dose of 2.5 dpa. We showed that in spite of the high degree of structural similarity, the formation of defects in these alloys is significantly different. In Cu-Y_2_O_3_, voids and dislocation loops were detected at all irradiation temperatures. The high density of voids in Cu-Y_2_O_3_ can be explained by the deoxidation with the aid of boron. Void swelling showed its maximum value at 350 °C. The dislocation loops were found to have an a_0_½⟨110⟩ Burges vector. Faulted loops with an a_0_1/3⟨111⟩ Burgers vector were not detected. In contrast, in the CuCrZrV alloy, the voids were formed in negligible amounts, while the number density of dislocation loops of the a_0_½⟨110⟩ type was the same as in ODS-Cu.

Neutron irradiation also affects the distribution of alloying and transmutation-induced elements in Cu. The EDX analysis revealed essential changes in the spatial distribution of precipitates in the CuCrCzV alloy. The size of Cr-rich particles increased and the number density remarkably decreased after irradiation at 350 °C and 450 °C. It was found that the Cr particles have a Bain orientation relationship ([001]_Cu-fcc_ ∥ [001]_Cr-bcc_, [100]_Cu-fcc_ ∥ [110]_Cr-bcc_) to the Cu matrix. In the sample irradiated at 450 °C, the number density of precipitates was reduced by two orders of magnitude. Contrary to this, the Y_2_O_3_ particles in the ODS-Cu alloy appear to have remained stable under all studied irradiation conditions. However, due to the inhomogeneous distribution of the Y_2_O_3_ particles in the investigated experimental alloy, slight modifications in size and density might have been remained undetected

It was also found that transmutation-induced Ni and Zn were mainly homogeneously distributed in the matrix. In some locations, there was a slight depletion of Zn along the grain boundaries in both alloys. Ni, however, showed an enrichment at the grain boundaries at 350 °C and 450 °C and a slight depletion at 150 °C.

The results show the limited stability of the CuCrZrV alloy under neutron irradiation at temperatures > 350 °C. The coarsening of the finely dispersed Cr particles led to a reduction in their strengthening effect, so that the mechanical properties should also degrade. The particles in the ODS-Cu alloys were not finely dispersed to prevent void formation. Additional technical steps are necessary to produce ODS alloys with finely dispersed Y_2_O_3_ particles that effectively reduce radiation attenuation.

## Figures and Tables

**Table 1:** Irradiation conditions for both CuCrZrV and Cu-Y_2_O_3_ alloys.

Irradiation Temperature (°C)	Damage Dose (dpa)
150	2.15
350	2.5
450	2.55

**Table 2:** Statistical data on radiation-induced defects in the CuCrZrV alloy.

T_irr_ (°C)	Void Size (nm)	Void Number Density ×10^20^ m^−3^	TEM Void Swelling (%)	Loop Size (nm)	Loop Number Density ×10^22^ m^−3^	Size of Cr Precipitates (nm)	Number Density of Cr Precipitates ×10^22^ m^−3^
unirr.	---	---	---	---	---	2.7 ± 0.15	4.8 ± 1.0
150	---	---	---	6 ± 1	15 ± 2	3.3 ± 0.2	3.3 ± 0.7
350	7.5 ± 2	8.0 ± 4.0	(1.5 ± 1) × 10^−2^	16 ± 2	21 ± 3	8.3 ± 0.5	0.32 ± 0.07
450	3 ± 1	1.0 ± 0.5	(2 ± 1.5) × 10^−4^	17 ± 2	7 ± 1	55 ± 10	0.03 ± 0.015

**Table 3:** Statistical data on radiation-induced defects in ODS-Cu alloys.

T_irr_ (°C)	Void Size (nm)	Void Number Density ×10^22^ m^−3^	TEM Void Swelling (%)	Loop Size (nm)	Loop Number Density ×10^22^ m^−3^
unirr.	11 ± 4	~0.04 ± 0.02	0.02 ± 0.01	---	---
150	5 ± 1.5	1.5 ± 0.4	0.17 ± 0.05	11 ± 1.0	1.5 ± 0.3
350	15.7 ± 4	0.33 ± 0.1	0.48 ± 0.15	16 ± 1.5	2.1 ± 0.4
450	7.8 ± 2	0.61 ± 0.15	0.18 ± 0.05	17 ± 1.5	5.3 ± 1.5

## Data Availability

The original contributions presented in the study are included in the article, further inquiries can be directed to the corresponding author.
